# Supplementary material for: An open-source framework for large-scale, flexible evaluation of biomedical text mining systems
Source: J Biomed Discov Collab. 2008 Jan 29;3:1. doi: 10.1186/1747-5333-3-1 (PMC2276192; doi:10.1186/1747-5333-3-1)
Supplement: Additional file 1 — Evaluation platform source code. The additional file contains the software evaluation framework discussed in this paper. The most current release can be downloaded from SourceForge.net [1]. See the accompanying README file for instructions on installation and use. Also included in the distribution is a collection of UIMA wrappers for some commonly used BioNLP tools and annotated corpora. [file 1747-5333-3-1-S1.zip › bionlp_uima_repository-1.0.1/docs/api/src-html/edu/uchsc/ccp/util/nlp/parser/corpus/IProLinkCorpus1Parser.html]

```
001    package edu.uchsc.ccp.util.nlp.parser.corpus;
002    
003    import java.io.BufferedReader;
004    import java.io.IOException;
005    import java.util.ArrayList;
006    import java.util.List;
007    import java.util.regex.Matcher;
008    import java.util.regex.Pattern;
009    
010    import edu.uchsc.ccp.util.nlp.GenericTextUtils;
011    import edu.uchsc.ccp.util.nlp.annotation.AnnotationSet;
012    import edu.uchsc.ccp.util.nlp.annotation.Annotator;
013    import edu.uchsc.ccp.util.nlp.annotation.TextAnnotation;
014    import edu.uchsc.ccp.util.nlp.document.GenericDocument;
015    import edu.uchsc.ccp.util.nlp.ei.xml.XMLEntityExtractor;
016    import edu.uchsc.ccp.util.nlp.mention.ClassMentionTypes;
017    
018    /**
019     * A parser for the iProLink corpus1.xml file.  This class outputs the original text and accompanying annotations for each corpus document. Titles and abstracts
020     * are outputted as separate documents.<br>
021     * <br>
022     * Output Classes:<br>
023     * <ul>
024     * <li>protein</li>
025     * <li>compound protein</li>
026     * <li>acronym</li>
027     * </ul>
028     * @author williamb
029     *
030     */
031    public class IProLinkCorpus1Parser extends CorpusParser {
032        public static final int DOCUMENT_COLLECTION_ID = 9;
033        
034        private final String PROTEIN_TAG = "protein";
035        private final String ACRONYM_TAG = "acronym";
036        private final String COMPOUND_PROTEIN_TAG = "compound-protein";
037        
038        private GenericDocument nextTitleDocument = null;
039        private GenericDocument nextAbstractDocument = null;
040        private boolean returnedTitleDocument = false;
041        private boolean returnedAbstractDocument = false;
042        
043        private BufferedReader br;
044        
045        private final Annotator annotator =  new Annotator(new Integer(11),"","iProLink","PIR");
046            private final AnnotationSet annotationSet = new AnnotationSet(new Integer(8),"iProLink prot/acr/cmpd Gold Annotations","<protein>, <acronym>, and <compound-protein> annotations extracted from the iProLink corpus1.xml file.");
047        
048        @Override
049        public void close() throws IOException {
050        }
051    
052        @Override
053        public boolean hasNext() {
054            /* if we have not yet returned the abstract document, then return true */
055            if (returnedTitleDocument && !returnedAbstractDocument) {
056                return true;
057            } else {
058                /* we have already returned the abstract document, so check to see if there are new title and abstract documents to return */
059                try {
060                    return hasNextDocument();
061                } catch (IOException e) {
062                    error("Error while parsing the IProLink Corpus 1 file.");
063                    e.printStackTrace();
064                    return false;
065                }    
066            }
067        }
068    
069        @Override
070        public GenericDocument next() {
071            if (!returnedTitleDocument) {
072                returnedTitleDocument = true;
073                return nextTitleDocument;
074            } else {
075                returnedAbstractDocument = true;
076                return nextAbstractDocument;
077            }
078        }
079        
080        public IProLinkCorpus1Parser(String corpus1XMLFile) throws IOException {
081            br = GenericTextUtils.openReader(corpus1XMLFile);
082        }
083        
084        
085        /**
086         * corpus1.xml has tags: protein, acronym, and compound-protein
087         * @param iProLinkCorpus1File
088         * @param connectionPropertiesFile
089         * @throws IOException
090         */
091        public boolean hasNextDocument() throws IOException {
092            returnedAbstractDocument = false;
093            returnedTitleDocument = false;
094            String line;
095    
096            Pattern idPattern = Pattern.compile("PMID:(\\d+)");
097            Pattern titlePattern = Pattern.compile("TI\\s+-\\s?(.*)");
098            Pattern abstractPattern = Pattern.compile("AB\\s+-\\s?(.*)");
099            Matcher m;
100    
101            String titleText = null;
102            String abstractText = null;
103            String id = null;
104            boolean inAbstract = false;
105            boolean inTitle = false;
106    
107            while ((line = br.readLine()) != null) {
108                if (line.startsWith("PMID")) {
109                    m = idPattern.matcher(line);
110                    if (m.find()) {
111                        id = m.group(1);
112                    } else {
113                        warn("ID expected but not found.");
114                    }
115                } else if (line.startsWith("TI  - ")) {
116                    m = titlePattern.matcher(line);
117                    if (m.find()) {
118                        titleText = m.group(1);
119                        inTitle = true;
120                        inAbstract = false;
121                    } else {
122                        warn("Title expected but not found.");
123                    }
124                } else if (line.startsWith("AB  - ")) {
125                    m = abstractPattern.matcher(line);
126                    if (m.find()) {
127                        abstractText = m.group(1);
128                        inAbstract = true;
129                        inTitle = false;
130                    } else {
131                        warn("Abstract expected but not found.");
132                    }
133                } else if ((line.startsWith(">") || line.trim().startsWith("</articleinfo>")) && (id != null)) {
134                    inAbstract = false;
135                    inTitle = false;
136    
137                    System.out.println("ID: " + id);
138                    System.out.println("TITLE: " + titleText);
139                    System.out.println("ABSTRACT: " + abstractText);
140                    
141                    
142                    /* search the titlefor entities */
143                    String documentID = id + "-t";
144    
145                    String titleWithProteins = titleText.replaceAll("</?" + ACRONYM_TAG + ">","").replaceAll("</?" + COMPOUND_PROTEIN_TAG + ">","");
146                    String titleWithAcronyms = titleText.replaceAll("</?" + PROTEIN_TAG + ">","").replaceAll("</?" + COMPOUND_PROTEIN_TAG + ">","");
147                    String titleWithCompoundProteins = titleText.replaceAll("</?" + PROTEIN_TAG + ">","").replaceAll("</?" + ACRONYM_TAG + ">","");
148                    
149                    List<TextAnnotation> proteinAnnotations = XMLEntityExtractor.parseEntitiesFromSparseUntokenizedXML(titleWithProteins, PROTEIN_TAG, PROTEIN_TAG, documentID, DOCUMENT_COLLECTION_ID, ClassMentionTypes.PROTEIN, annotator, annotationSet);
150                    List<TextAnnotation> acronymAnnotations = XMLEntityExtractor.parseEntitiesFromSparseUntokenizedXML(titleWithAcronyms, ACRONYM_TAG, ACRONYM_TAG, documentID, DOCUMENT_COLLECTION_ID, ClassMentionTypes.PROTEIN_ACRONYM, annotator, annotationSet);
151                    List<TextAnnotation> compoundProteinAnnotations = XMLEntityExtractor.parseEntitiesFromSparseUntokenizedXML(titleWithCompoundProteins, COMPOUND_PROTEIN_TAG, COMPOUND_PROTEIN_TAG, documentID, DOCUMENT_COLLECTION_ID, ClassMentionTypes.PROTEIN_COMPOUND, annotator, annotationSet);
152                    
153                    List<TextAnnotation> titleAnnotations = new ArrayList<TextAnnotation>();
154                    titleAnnotations.addAll(proteinAnnotations);
155                    titleAnnotations.addAll(acronymAnnotations);
156                    titleAnnotations.addAll(compoundProteinAnnotations);
157                    
158                    /* store the untagged title text */
159                    /* remove html tags from the title text */
160                    titleText = titleText.replaceAll(GenericTextUtils.escapeCharacterForRegEx("<" + PROTEIN_TAG + ">"), "").replaceAll(GenericTextUtils.escapeCharacterForRegEx("</" + PROTEIN_TAG + ">"), "");
161                    titleText = titleText.replaceAll(GenericTextUtils.escapeCharacterForRegEx("<" + ACRONYM_TAG + ">"), "").replaceAll(GenericTextUtils.escapeCharacterForRegEx("</" + ACRONYM_TAG + ">"), "");
162                    titleText = titleText.replaceAll(GenericTextUtils.escapeCharacterForRegEx("<" + COMPOUND_PROTEIN_TAG + ">"), "").replaceAll(GenericTextUtils.escapeCharacterForRegEx("</" + COMPOUND_PROTEIN_TAG + ">"), "");
163                    
164                    /* create two new Generic Documents, one for the title and one for the abstract */
165                    nextTitleDocument = new GenericDocument();
166                    nextTitleDocument.setDocumentText(titleText);
167                    nextTitleDocument.setAnnotations(titleAnnotations);
168                    nextTitleDocument.setDocumentID(id + "-t");
169                    
170                    /* now search the abstract */
171                    documentID = id + "-a";
172                    
173                    String abstractWithProteins = abstractText.replaceAll("</?" + ACRONYM_TAG + ">","").replaceAll("</?" + COMPOUND_PROTEIN_TAG + ">","");
174                    String abstractWithAcronyms = abstractText.replaceAll("</?" + PROTEIN_TAG + ">","").replaceAll("</?" + COMPOUND_PROTEIN_TAG + ">","");
175                    String abstractWithCompoundProteins = abstractText.replaceAll("</?" + PROTEIN_TAG + ">","").replaceAll("</?" + ACRONYM_TAG + ">","");
176                    
177                    proteinAnnotations = XMLEntityExtractor.parseEntitiesFromSparseUntokenizedXML(abstractWithProteins, PROTEIN_TAG, PROTEIN_TAG, documentID, DOCUMENT_COLLECTION_ID, ClassMentionTypes.PROTEIN, annotator, annotationSet);
178                    acronymAnnotations = XMLEntityExtractor.parseEntitiesFromSparseUntokenizedXML(abstractWithAcronyms, ACRONYM_TAG, ACRONYM_TAG, documentID, DOCUMENT_COLLECTION_ID, ClassMentionTypes.PROTEIN_ACRONYM, annotator, annotationSet);
179                    compoundProteinAnnotations = XMLEntityExtractor.parseEntitiesFromSparseUntokenizedXML(abstractWithCompoundProteins, COMPOUND_PROTEIN_TAG, COMPOUND_PROTEIN_TAG, documentID, DOCUMENT_COLLECTION_ID, ClassMentionTypes.PROTEIN_COMPOUND, annotator, annotationSet);
180              
181                    //System.err.println("DEBUG -- IProLinkParser: Found annotations in abstract: protein" + proteinAnnotations.size() + " acronym: " + acronymAnnotations.size() + " compound: " + compoundProteinAnnotations.size());
182                    
183                    /* store the untagged abstract text */
184                    /* remove html tags from the abstract text */
185                    abstractText = abstractText.replaceAll(GenericTextUtils.escapeCharacterForRegEx("<" + PROTEIN_TAG + ">"), "").replaceAll(GenericTextUtils.escapeCharacterForRegEx("</" + PROTEIN_TAG + ">"), "");
186                    abstractText = abstractText.replaceAll(GenericTextUtils.escapeCharacterForRegEx("<" + ACRONYM_TAG + ">"), "").replaceAll(GenericTextUtils.escapeCharacterForRegEx("</" + ACRONYM_TAG + ">"), "");
187                    abstractText = abstractText.replaceAll(GenericTextUtils.escapeCharacterForRegEx("<" + COMPOUND_PROTEIN_TAG + ">"), "").replaceAll(GenericTextUtils.escapeCharacterForRegEx("</" + COMPOUND_PROTEIN_TAG + ">"), "");
188                                    
189                    List<TextAnnotation> abstractAnnotations = new ArrayList<TextAnnotation>();
190                    abstractAnnotations.addAll(proteinAnnotations);
191                    abstractAnnotations.addAll(acronymAnnotations);
192                    abstractAnnotations.addAll(compoundProteinAnnotations);
193                    
194                    nextAbstractDocument = new GenericDocument();
195                    nextAbstractDocument.setDocumentText(abstractText.trim());
196                    nextAbstractDocument.setAnnotations(abstractAnnotations);
197                    nextAbstractDocument.setDocumentID(id + "-a");
198    
199                    return true;
200                } else {
201                    /* there are line breaks in the abstract, so if we are in the abstract, we add the line to the abstract text */
202                    if (inAbstract) {
203                        abstractText += (" " + line.trim());
204                    } else if (inTitle) {
205                        titleText += (" " + line.trim());
206                    } else {
207                        warn("Unexpected tag at beginning of line: " + line);
208                    }
209                }
210            }
211            
212            return false;
213            
214            
215        }
216    
217    }
```
